# Combinations of Polymorphic Markers of Chemokine Genes, Their Receptors and Acute Phase Protein Genes As Potential Predictors of Coronary Heart Diseases

**Published:** 2016

**Authors:** T.R. Nasibullin, L.F. Yagafarova, I.R. Yagafarov, Y.R. Timasheva, V.V. Erdman, I.A. Tuktarova, O.E. Mustafina

**Affiliations:** Institute of Biochemistry and Genetics, Ufa Research Center of the Russian Academy of Sciences, Ufa, the Republic of Bashkortostan, Prospect Octyabrya, 71, 450054, Russia; Medical and sanitary unit of PJSC “Tatneft” and the city of Almetyevsk, Almetyevsk, the Republic of Tatarstan, Radischeva Str., 67, 423450, Russia

**Keywords:** coronary heart disease, genetic polymorphism, complex traits, APSampler

## Abstract

Atherosclerosis, the main factor in the development of coronary heart diseases (CHD), is an inflammatory response to endothelial layer damage in the arterial bed. We have analyzed the association between CHD and the polymorphic markers of genes that control the synthesis of proteins involved in the processes of adhesion and chemotaxis of immunocompetent cells: rs1024611 (–2518A>G, *CCL2 *gene), rs1799864 (V64I, *CCR2* gene), rs3732378 (T280M, *CX3CR1 *gene), rs1136743 (A70V, *SAA1 *gene), and rs1205 (2042C>T, *CRP *gene) in 217 patients with CHD and 250 controls. Using the Monte Carlo method and Markov chains (APSampler), we revealed a combination of alleles/genotypes associated with both a reduced and increased risk of CHD. The most significant alleles/genotypes areSAA1*T/T+CRP*C+CX3CR1*G/A (*P*_perm_ = 0.0056, OR = 0.07 95%CI 0.009–0.55),SAA1*T+CRP*T+CCR2*G/A+CX3CR1*G (*P*_perm_ = 0.0063, OR = 14.58 95%CI 1.88–113.04), SAA1*T+CCR2*A+CCL2* G/G (*P*_perm_ = 0.0351, OR = 10.77 95%CI 1.35–85.74).

## INTRODUCTION

Coronary heart diseases (CHD) and the main factor of their development, atherosclerosis, are among the most frequent causes of disability and mortality in the majority of developed countries. The molecular and genetic basis of hereditary predisposition to CHD is actively studied, and one of the important directions in such research is the analysis of the association between polymorphic DNA markers and the disease. Genome-wide association studies (GWAS) using high-density microarrays and analysis of individual polymorphic markers located in the regions of the genes encoding for products involved in the pathogenesis of the disease (candidate genes) are used for this purpose. Most studies analyze the contribution of individual polymorphic markers in the formation of hereditary predisposition to the disease. At the same time, since atherosclerosis, with the exception of rare monogenic variants, is a multifactorial polygenic disease caused by complex interactions between genetic and environmental factors, the analysis of the combinations of factors determining the activity of individual pathogenetic links appears to be more promising.

According to modern concepts, atherosclerotic lesion of the vascular wall is caused by an inflammatory reaction developing in response to damage to vascular endothelium [1]. The inflammatory process at all stages of atherosclerosis involves immune cells , the participation of which in endothelial damage includes their mobilization from the bone marrow, adhesion, chemotaxis, transformation, change in the ratio of different leukocyte subclasses , etc. All these processes are controlled by a variety of proteins and inflammatory mediators, including chemokines and acute-phase proteins.

Chemokines are a group of low-molecular-weight cytokines the primary function of which is mediating the migration of various cells expressing chemokine receptors from the bloodstream to inflammation or tumor sites. Chemokine CCL2 (monocyte chemoattractant protein-1, MCP-1) and its receptor CCR2 play a central role in monocyte chemotaxis and infiltration of vascular wall as shown in the experiments in mice that demonstrated enhanced expression of *CCR2 *gene on the surface of monocytes and increased synthesis of CCL2 in hyperlipidemic conditions [2, 3]. Chemokine CX3CL1 (fractalkine) exists in two forms: a membrane-bound from, providing the adhesion of leukocytes to endothelium of blood vessels, and a soluble form acting as chemoattractant [4]. CX3CL1 interacts with CX3CR1 receptor found on the membranes of T-lymphocytes, monocytes, dendritic cells, natural killer cells, and smooth muscle cells, thereby providing their migration, adhesion, and proliferation [5].

C-reactive protein (CRP) and serum amyloid A (SAA) are major acute phase inflammatory proteins . During the first hours of injury, their level increases by 20-100 times, in some cases by 1,000 times and more, which makes these proteins universal markers of acute inflammatory response. It has been demonstrated *in vitro *that CRP is capable of inducing the expression of adhesion molecules and chemokine CCL2 in endothelial cells [6, 7]. SAA also promotes the migration of monocytes and lymphocytes, increasing chemokine expression [8].

The aim of our study was to analyze the contribution of combinations of the polymorphic markers rs1024611 (-2518A>G, *CCL2 *gene), rs1799864 (V64I, *CCR2 *gene), rs3732378 (T280M, *CX3CR1 *gene), rs1136743 (A70V,* SAA1 *gene), and rs1205 (2042C>T, *CRP *gene) to the formation of genetic predisposition to CHD.

## EXPERIMENTAL

The study group consisted of unrelated male patients (*N *= 217) with verified diagnosis of CHD (165 cases of myocardial infarction, 52 cases of functional class 3–4 cardiac angina) treated in the Medical and Sanitary Unit of PJSC Tatneft in the city of Almetyevsk. Coronary artery atherosclerosis was confirmed by angiography. The average age of patients at enrollment in the study was 53.55 ± 5.78 years. Patients with diabetes and other endocrine disorders were excluded from the study. The control group consisted of unrelated sexand age-matched (average age 50.48 ± 6.03) individuals (*N *= 250). All members of the control group did not have any signs of cardiovascular disease according to their medical history, clinical examination and electrocardiography. All participants in the study belonged to the Tatar ethnic group. All participants gave their informed consent.

**Table 1:** List of the polymorphic markers included in the study, their localization, primer sequences, restriction enzymes, and allele nomenclature

Gene, chromosome localization	Polymorphism, localization	Primer sequence, restriction enzyme	Allele, fragment size, bp
CCL2 17q12	rs1024611 –2518A>G 5’-end	F 5’-ctc acg cca gca ctg acc tcc-3’ R 5’-agc cac aat cca gag aag gag acc-3’ PvuII	A – 300 G – 228 and 72
CCR2 3p21.31	rs1799864 V64I exon 2	F 5’-tgc ggt gtt tgt gtt gtg tgg tca-3’ R 5’-aga tgg cca ggt tga gca ggt-3’ FokI	G(V) – 282 and 74 A(I) – 198, 84 and 74
CX3CR1 3p21.3	rs3732378 T280M exon 2	F 5’-gga ctg agc gcc cac aca gg-3’ R 5’-agg ctg gcc ctc agt gtg act-3’ Alw26I	A(M) – 148 G(T) – 128 and 20
SAA1 11p15.1	rs1136743 A70V exon 3	F 5’-ccc ctc taa ggt gtt gtt gga-3’ R 5’-ctc cac aag gag ctc gtc tc-3’ BshNI	T(V) – 289 C(A) – 183 and 106
CRP 1q23.2	rs1205 2042C>T 3’-untranslated region	F 5’-aga aaa cag ctt gga ctc act ca-3’ R 5’-tga gag gac gtg aac ctg gg-3’ C 5’-cca gtt tgg ctt ctg tcc tca c-3’ T 5’-cca gtt tgg ctt ctg tcc tca t-3’	IC* – 235 Allele – 82

*IC – internal control containing studied mutation

DNA samples were isolated from peripheral blood leukocytes by phenol-chloroform extraction [9]. All polymorphic markers, except for rs1205, were genotyped by polymerase chain reaction (PCR), followed by treatment of the amplification products with the appropriate restriction enzyme. Polymorphic marker rs1205 was genotyped by site-specific PCR. Amplicons were separated by electrophoresis in 7% polyacrylamide or 2% agarose gel. Primers and restriction enzymes specific to each marker were selected using the DNASTAR 5.05 software package and http://www. ncbi.nlm.nih.gov/snp database. Primer sequences, restriction enzymes, and the resulting fragment lengths are presented in *Table 1*.

The frequencies of the genotypes and alleles of polymorphic markers in the study groups were compared using two-tailed Fisher’s exact test. Compliance of the observed genotype frequency distribution to the theoretically expected one under Hardy-Weinberg equilibrium was determined using an exact test implemented in the Arlequn 3.0 software. The search for combinations of alleles/genotypes associated with CHD was performed using APSampler 3.6.1 software available here: https://code.google.com/p/apsampler. The basic algorithm of this software is described elsewhere [10]. Permutation test was used as correction for multiplecomparisons, and P_perm_ < 0.05 was considered statistically significant.

## RESULTS AND DISCUSSION

The results of the analysis of the genotype and allele frequency distribution for the studied polymorphic markers are shown in *Table 2*. The distribution of the genotype frequencies of polymorphic markers in the control group was in agreement with the theoretically expected ones according to the Hardy-Weinberg equilibrium. A comparative analysis of genotype frequency distribution demonstrated that CRP*T/T (*P *= 0.02, OR = 1.74 95%CI 1.1–2.75) and SAA1*T/C (*P *= 0.014, OR = 1.61 95%CI 1.11–2.34) genotype frequencies were increased in the group of patients with CHD.

**Table 2:** Results of the analysis of association between polymorphic DNA markers and the risk of coronary heart disease

Genotype/ Allele	Control, %		Cases, %		P
	n	Frequency (95%CI)	n	Frequency (95%CI)	
CRP rs1205 (2042C > T)					
*C/C	83	33.2 (27.39–39.41)	57	26.27 (20.54–32.65)	0.1065
*C/T	127	50.8 (44.43–57.16)	106	48.85 (42.02–55.71)	0.7108
*T/T	40	16 (11.68–21.14)	54	24.88 (19.28–31.19)	0.0205
*C	293	58.6 (54.14–62.96)	220	50.69 (45.88–55.49)	0.0176
*T	207	41.4 (37.04–45.86)	214	49.31 (44.51–54.12)	
SAA1 rs1136743 (A70V)					
*C/C	71	28.4 (22.9–34.42)	48	22.12 (16.78–28.24)	0.1363
*T/C	135	54 (47.61–60.3)	142	65.44 (58.7–71.75)	0.0141
*T/T	44	17.6 (13.09–22.9)	27	12.44 (8.36–17.58)	0.1549
*C	277	55.4 (50.92–59.81)	238	54.84 (50.02–59.59)	0.8951
*T	223	44.6 (40.19–49.08)	196	45.16 (40.41–49.98)	
CX3CR1 rs3732378 (T280M)					
*C/C	162	64.8 (58.53–70.71)	141	64.98 (58.23–71.31)	1
*C/T	80	32 (26.26–38.17)	67	30.88 (24.8–37.48)	0.8418
*T/T	8	3.2 (1.39–6.21)	9	4.15 (1.91–7.73)	0.6272
*C	404	80.8 (77.07–84.16)	349	80.41 (76.36–84.05)	0.9339
*T	96	19.2 (15.84–22.93)	85	19.59 (15.95–23.64)	
CCL2 rs1024611 (–2518A > G)					
*A/A	151	60.4 (54.04–66.51)	126	58.06 (51.2–64.71)	0.6373
A/G	82	32.8 (27.02–39)	70	32.26 (26.09–38.92)	0.9213
*G/G	17	6.8 (4.01–10.66)	21	9.68 (6.09–14.41)	0.3092
*A	384	76.8 (72.85–80.43)	322	74.19 (69.81–78.25)	0.3605
*G	116	23.2 (19.57–27.15)	112	25.81 (21.75–30.19)	
CCR2 rs1799864 (V64I)					
*G/G	181	72.4 (66.41–77.85)	157	72.35 (65.89–78.19)	1
*G/A	61	24.4 (19.21–30.21)	55	25.35 (19.7–31.68)	0.8306
*A/A	8	3.2 (1.39–6.21)	5	2.3 (0.75–5.29)	0.5884
*G	423	84.6 (81.13–87.65)	369	85.02 (81.31–88.25)	0.9272
*A	77	15.4 (12.35–18.87)	65	14.98 (11.75–18.69)	

A total of 743 combinations of genotypes and alleles associated with CHD were identified using the APSampler algorithm; after the validation of the results, five combinations remained associated with a reduced risk of CHD, and seven combinations were associated with an increased risk of CHD (*Table 3*).

**Table 3:** Combinations of the alleles/genotypes associated with a coronary heart disease obtained using the APSampler algorithm

Combination	Frequency, %		P_perm_	OR	95%CI_OR_
	Control	Cases			
SAA1*T/T+CRP*C+CX3CR1*G/A	6.00	0.46	0.0056	0.07	0.009–0.55
SAA1*T+CX3CR1* G/A	7.30	0.92	0.0056	0.12	0.03–0.56
SAA1*T+CRP*T+CCR2*G/A+CX3CR1*G	0.40	5.53	0.0063	14.58	1.88–113.04
SAA1*T/C+CCR2*G+CCL2*G	19.60	30.41	0.0348	1.79	1.17–2.74
SAA1*T+CCR2*A+CCL2*G/G	0.40	4.15	0.0351	10.77	1.35–85.74
SAA1*T+CRP*T+CCR2*A+CX3CR1*A	1.20	5.53	0.039	4.82	1.34–17.31
SAA1*T+CRP*T/T	12.40	21.20	0.0393	1.9	1.16–3.12
CRP*T/T+CCR2*A+CCL2*G	0.80	4.61	0.0425	5.99	1.3–27.65
CRP*T+CCR2*G/A+CX3CR1*A	2.40	7.37	0.0436	3.24	1.24–8.43
SAA1*T/T+CRP*T+CCL2*A	16.80	10.15	0.049	0.5	0.29–0.89
CRP*C+CCL2*A	78.40	68.66	0.0492	0.6	0.4–0.92
SAA1*T/T+CX3CR1*G+CCL2*A	20.19	9.22	0.0492	0.5	0.29–0.89

In the current study, we analyzed the frequency distribution of genotypes and alleles of polymorphic markers of *SAA1*, *CRP*, *CCL2*, *CCR2, *and *CX3CR1 *genes in the ethnically homogeneous groups of men with CHD and control subjects. Combinations of polymorphic markers associated with the risk of disease development were identified using the APSampler software. It should be noted that while statistically significant results were obtained only for the *SAA1 *and *CRP *genes when the studied polymorphic markers were analyzed individually, the identified patterns included all the studied polymorphic markers in variouscombinations.

Allele SAA1*T (rs1136743) is present in the combinations associated with both an increased and reduced risk of CHD. As shown by other authors, homozygous carriers of rs1136743*T/rs1136747*C haplotype in a Moscow population of patients with rheumatoid arthritis and patients with Mediterranean fever in Turkey have an increased risk of amyloidosis [11, 12]. As mentioned earlier, SAA stimulates the expression of proinflammatory chemokines [8]. In addition, SAA is capable of replacing apolipoprotein A in high-density lipoproteins (HDL), resulting in a loss of antiatherogenic properties of HDL and its conversion into the proatherogenic form [13]. At the same time, there is evidence of the antiatherogenic effect of SAA. In particular, SAA inhibits platelet activation and prevents their aggregation at sites of endothelial injury [14]; it has been reported that SAA promotes the removal of HDL from the cell [15].

According to the results of a multicenter study which included patients with myocardial infarction (MI) from six European cities, the carriers of* CRP *rs1205 *T/T genotype have a lower CRP plasma level compared to carriers of the CRP*C allele [16]. Similar results were obtained for a Reykjavik population [17], European Americans, and African Americans [18]. At the same time, Hatzis G. *et al*. demonstrated the association of CRP*T/T genotype with an increased risk of coronary atherosclerosis in a Greek population [19], the association of CRP*T allele with an increased risk of vascular complications in patients with type 2 diabetes was also reported [20]. According to the results of several studies, CRP stimulates the expression of adhesion molecules (VCAM1, ICAM1 and selectin E) [6], CCL2 chemokine [7], and inhibits the production of nitric oxide [21] in endothelial cells, while no evidence of proatherogenic properties of CRP was obtained from animal models. In *APOE *and *LDLR *knockout mice, the knockout of the *CRP *gene did not lead to a significant reduction in the size of atherosclerotic vascular lesions [22], and the introduction of human CRP in *LDLR*-/- mice had no significant effect [23]. Moreover, there is evidence of antiatherogenic properties for CRP: Lei Z.B. *et al*. demonstrated its ability to bind to oxidized low-density lipoproteins (LDL) [24], which, in turn, upregulate the expression of chemokines and adhesion molecules [25-27].

Our data on the role of rs1024611 (*CCL2 *gene) and rs1799864 (*CCR2 *gene) polymorphic markers in the formation of a genetic predisposition to CHD are consistent with the results of other studies. For example, the association of CCL2*G allele with ischemic stroke has been demonstrated in the American population [28]. According to a meta-analysis of the results of 21 studies, carriage of CCL2*G allele is associated with increased risk of CHD among Europeans [29]. The association of CCR2*G/A genotype with abdominal aortic aneurysm has been shown in the Turkish population [30], while in the Czech Republic the same genotype is a marker of the risk of MI in women under the age of 50 [31]. A number of studies have established a correlation between the CCL2*G/G genotype and higher plasma levels of CCL2 [32, 33], as well as with increased* CCL2 *expression compared to CCL2*A allele carriers [34]. Furthermore, we have found an association of CCL2*G/G genotype with an increased risk of MI, and an association of CCL2*G/G+CCR2*A combination with an increased risk of essential hypertension among the Tatars of Bashkortostan [35, 36].

The role of the rs3732378 (*CX3CR1 *gene)polymorphic marker is controversial. McDermott *et al*. demonstrated association between CX3CR1*M allele and lower rates of cell adhesion and leukocyte chemotaxis, and decreased risk of CHD[37]. At the same time, CX3CR1*M allele was found to be associated with type 2 diabetes among European Americans [38]. According to the results of meta-analysis of 49 studies, CX3CR1*T/M genotype is associated with decreased risk of atherosclerosis and CHD, while CX3CR1*M/M genotype is associated with increased risk of ischemic cerebrovascular disease [39], which is consistent with our data.

## CONCLUSIONS

In conclusion, it should be noted that our results support the hypothesis of the influence of the *SAA1*, *CRP*,* CCL2*, *CCR2, *and *CX3CR1 *gene polymorphisms on the processes that play an important role in CHD pathogenesis. We also demonstrated that allelic variants of the *SAA1 *and *CRP *genes can have both a negative and positive effect on the development of the disease depending on the genetic environment, which illustrates the thesis of a complex nonlinear interaction of the studied factors and does not contradict the results obtained in other studies.

## Abbreviations

CHD: coronary heart disease
MI: myocardial infarction
CRP: C-reactive protein
SAA: serum amyloid A
